# Guidelines for contributors

**DOI:** 10.2471/BLT.25.960125

**Published:** 2025-01-01

## 1. Scope and editorial policy

### 1.1 Content

The mission of the *Bulletin of the World Health Organization* (WHO) is “to publish and disseminate scientifically rigorous public health information of international significance that enables policy-makers, researchers and practitioners to be more effective; it aims to improve health, particularly among vulnerable populations.” The *Bulletin *is fully open-access and charges no author fees. 

The *Bulletin *welcomes a variety of unsolicited manuscripts (see below, 1.1.1.). These are initially screened in-house for originality, relevance to an international public health audience and scientific rigour. If manuscripts pass the initial screening, they are sent to peer reviewers, whose opinions are considered by the journal’s editorial advisers upon deciding whether to invite a revision. Several rounds of revision and review may be requested before a paper is accepted. All accepted papers are subject to editorial revision, which may involve substantive changes, shortening or restructuring the text and deleting superfluous tables and figures. Please also note that the title of every paper accepted for publication in the *Bulletin* is edited for clarity and to align with WHO’s style guide. The word limits given for each type of manuscript do not include the abstract (where applicable), tables, boxes, figures and references or appendices, if any. Authors of accepted papers must upload any supplementary material to a data repository that provides a digital object identifier (doi). The main types of manuscripts are outlined below.

#### 1.1.1. Unsolicited manuscripts

We welcome unsolicited submissions to the Research, Systematic reviews, Policy & practice, Lessons from the field and Perspectives sections of the *Bulletin.* All manuscripts intended for the first four of these sections must include two paragraphs highlighting what the submission adds to the literature. The paragraphs should briefly explain what was already known about the topic concerned, and what new knowledge the manuscript contributes.

We only consider papers that have not been published – in full or in part –elsewhere. However, an exception is made for pre-prints of research papers in the context of a public health emergency of international concern. 

##### Research

Methodologically rigorous research of relevance to international public health. Formal scientific presentations having not more than 3000 words and 50 references, plus a structured abstract (see below, 2.7); peer reviewed. As clear reporting is needed for readers and reviewers when judging the quality of research, studies should comply with the relevant reporting guidelines, available on the EQUATOR Network website: http://www.equator-network.org/about-us/uk-equator-centre/equator-publications/equator-network-publications-2010/. Approval or exemption by the relevant ethical review committee must be reported in the manuscript. Operational and implementation research should be reported in compliance with pertinent guidelines (available at: https://pubmed.ncbi.nlm.nih.gov/26769997/). Intervention trials as defined by WHO (i.e. “any research study that prospectively assigns human participants or groups of humans to one or more health-related interventions to evaluate the effects on health outcomes”) require registration in a public trials registry acceptable to the International Committee of Medical Journal Editors (ICMJE) before submission, and the registration number must be provided at the end of the abstract. Acceptable registries are listed at: https://www.who.int/clinical-trials-registry-platform/network. For papers reporting primary research, at least one author must be affiliated with an institution in the country in which the primary research was conducted. Results from human subject research should be disaggregated by age and sex and/or gender, in compliance with the SAGER guidelines: https://researchintegrityjournal.biomedcentral.com/articles/10.1186/s41073-016-0007-6/tables/1

##### Systematic reviews 

Exhaustive, critical assessments of published and unpublished studies on research questions concerning interventions, policies or practices in public health, with meta-analysis when feasible. The systematic review protocol should be registered with the International Prospective Register of Systematic Reviews (PROSPERO) or another similar registry. Not more than 3000 words plus a structured abstract (see below, 2.7) and with a number of references concordant with the scope of the review; peer reviewed. Steps leading to inclusion and exclusion of studies should be illustrated in a flow diagram. The full search strategy should be provided in a box. Authors should follow the reporting guidelines for systematic reviews and meta-analyses (PRISMA) available at: https://www.equator-network.org/reporting-guidelines/prisma/. The *Bulletin* requires that searches are not restricted to a particular language, and the methods used to analyse multilingual content must be reported. The search must have been done within six months of the date of submission.

##### Policy & practice

Analytical assessments, critical policy analysis, debates or hypothesis-generating papers on public health topics; not more than 3000 words and 50 references, plus a non-structured abstract (see below, 2.7); peer reviewed.

##### Lessons from the field

Papers that capture experiences and practice gained in solving specific public health problems in low- and middle-income countries. Convincing evidence of effect should be provided. Not more than 1500 words and 15 references, plus a structured abstract (see below, 2.7); not more than one table and one figure; must include one box listing at least three lessons learnt, including any challenges and how/if these were overcome; peer reviewed (please see: https://www.ncbi.nlm.nih.gov/pmc/articles/PMC2626521/for more details on the elements required in Lessons from the field manuscripts) Operational and implementation research reports should follow the specifications described above for the research section. 

##### Perspectives

Views, hypotheses or discussions (with a clear message) surrounding an issue of public health interest. No abstract, up to 1500 words, no more than 12 references; peer reviewed.

#### 1.1.2. Commissioned manuscripts

*Bulletin* editors occasionally commission manuscripts from an author group on a specific topic. In this instance, *Bulletin* editors, guided by editorial advisors, will provide as much editorial support as feasible to authors to develop a manuscript. However, commissioning a paper does not guarantee that a paper will be sent for peer review or that it will eventually be accepted or published. The *Bulletin* will not publish commissioned papers that do not meet WHO’s requirements. Editorials are normally commissioned by the editors. Editorials published in the *Bulletin *are limited to 800 words with no more than 12 references. Authors wishing to submit an unsolicited editorial should first contact the *Bulletin* office (see below, 2.1).

### 1.2 Ethical issues

WHO publishes the results of research involving human subjects only if fully compliant with ethical principles, including the provisions of the World Medical Association Declaration of Helsinki (as amended by the 59th General Assembly, Seoul, the Republic of Korea, October 2008; available at: https://www.wma.net/wp-content/uploads/2016/11/DoH-Oct2008.pdf) and with the additional requirements, if any, of the country in which the research was carried out. Any manuscript describing the results of such research must contain a clear statement to this effect and should specify that the free and informed consent of the subjects or their legal guardians was obtained and that the relevant institutional or national ethics review board approved the investigation. WHO Research Ethics Review Committee clearance is required for papers that report research supported by WHO or that are authored or co-authored by someone who was a WHO staff member when the research was conducted.

The *Bulletin* is a member of the Committee on Publication Ethics (COPE; see: https://publicationethics.org). Issues involving publication ethics may be referred to this committee by the editors. 

### 1.3 Competing interests

A competing interest arises when a professional judgement concerning a primary interest (such as patients’ welfare or the validity of research) may be influenced by a secondary interest (such as financial gain or professional rivalry). We ask all authors to disclose at the time of submission any competing interests that they may have. Examples of competing interests may be found at: https://www.icmje.org. Further information on competing interests is available at: https://www.ncbi.nlm.nih.gov/pmc/articles/PMC2626344/. 

### 1.4 Funding

Authors should identify the funding sources of the work undertaken, affirm that they have not entered into an agreement with the funder that may have limited their ability to complete the research as planned, and indicate that they have had full control of all primary data.

### 1.5 Appeals process

Authors of rejected papers can appeal the decision by following the procedures outlined in https://www.ncbi.nlm.nih.gov/pmc/articles/PMC2626344/. Appeals should be received within four weeks of rejection.

## 2. Preparation and submission of manuscripts

### 2.1 Correspondence

Manuscripts should be submitted to the *Bulletin *via our submissions website (https://submit.bwho.org), where full instructions are given. Queries about online submissions should be sent to the editorial office: bulletin.submit.ask@who.int. Authors requiring assistance with online submission can contact the editorial office.

### 2.2 Uniform requirements

Manuscripts should be prepared in accordance with the *ICMJE recommendations for the conduct, reporting, editing and publication of scholarly work in medical journals*. The complete document is available at: https://www.icmje.org/recommendations/. 

### 2.3 Languages

Manuscripts should be submitted in English and will be published in that language in the *Bulletin*; the abstracts are translated into Arabic, Chinese, French, Russian and Spanish, at no cost to authors.

### 2.4 Authorship

On the manuscript’s title page, authors should give their full names and the name, city and country of their institutions, as well as their institutional affiliation at the time of writing and ORCID number. The corresponding author must also provide a full postal address, which will be published with their email address unless otherwise requested. If authors wish to share first authorship, this information will be contained in the acknowledgements section. Academic titles and positions are not required. If an author has several affiliations, only the primary institution should be provided. Other institutions may be noted in the acknowledgements section. The criteria for authorship described in the *ICMJE recommendations* (see above, 2.2) must be rigorously observed. Each author should have participated sufficiently in the work being reported to take public responsibility for the paper’s content and each author’s contribution should be described in the online submission system (not within the manuscript itself). The *Bulletin* encourages submissions from authors in low- and middle-income countries, and in line with this policy at least one author should have a professional affiliation in the country where the study was conducted.

### 2.5 Licence for publication

The *Bulletin *is a fully open-access journal and charges no author fees. Authors are responsible for obtaining permission to reproduce in their articles any material under copyright protection. They should send the letter granting such permission to the editorial office when they submit their papers.

On submission, the corresponding author must indicate agreement on behalf of all authors that their submission, if accepted, will be published under the intergovernmental organizations’ creative commons attribution licence (CCBY 3.0 IGO). 

### 2.6 Figures, tables and boxes

These should be used only to enhance the understanding of the text, not to repeat what can be clearly communicated within the text. All figures, tables and boxes should be numbered consecutively (e.g. Fig. 1, Table 1 and Box 1).

### 2.7 Abstracts

Abstracts should highlight the text’s most important points and should be provided in English for the following types of papers: Research, Systematic reviews, Policy & practice, and Lessons from the field. The abstract should not exceed 250 words. It will be translated into Arabic, Chinese, French, Russian and Spanish after the paper is accepted. Structured abstracts are required for Research papers and Systematic reviews (Objective, Methods, Findings, Conclusion) and for Lessons from the field papers (Problem, Approach, Local setting, Relevant changes, Lessons learnt). 

### 2.8 Bibliographic references

Reference citations should be numbered in parentheses or superscript consecutively as they occur in the text, and references should be listed in accordance with the *ICMJE recommendations* *for the conduct, reporting, editing and publication of scholarly work in medical journals * (https://www.icmje.org/icmje-recommendations.pdf). The accuracy and completeness of all references is the authors’ responsibility, and authors are also responsible for dating access to URLs, providing the year, month and day of when they were accessed.

### 2.9 Maps

Papers should contain no maps unless an important finding cannot be conveyed without them or unless they are needed to make an essential point. Maps that show international borders, partially or in full, must adhere to WHO guidelines following these standard operating procedures: https://gis-who.hub.arcgis.com/pages/sops-for-maps. Maps can be created by the WHO GIS Centre for Health. Submit a map request at https://gis-who.hub.arcgis.com/pages/map-request-page at least one week before the map is needed. 

### 2.10 Supplementary material

The print version of the *Bulletin* may refer to online material that supplements articles and is made available on the *Bulletin’s* website. Additional supplementary material may be hosted on sites managed by the authors’ institutions; digital object identifiers for these locations need to be provided by the authors. 

